# Diverse infectivity, transmissibility, and pathobiology of clade 2.3.4.4 H5Nx highly pathogenic avian influenza viruses in chickens

**DOI:** 10.1080/22221751.2023.2218945

**Published:** 2023-06-12

**Authors:** Jung-Hoon Kwon, Kateri Bertran, Dong-Hun Lee, Miria Ferreira Criado, Lindsay Killmaster, Mary J. Pantin-Jackwood, David E. Swayne

**Affiliations:** a Agricultural Research Service, U.S. Department of Agriculture, U.S. National Poultry Research Center, Athens, GA, USA; b College of Veterinary Medicine, Kyungpook National University, Daegu, Republic of Korea; c Unitat mixta d’Investigació IRTA-UAB en Sanitat Animal, Centre de Recerca en Sanitat Animal (CReSA), Bellaterra, Spain; d IRTA. Programa de Sanitat Animal, Centre de Recerca en Sanitat Animal (CReSA), Bellaterra, Spain; e College of Veterinary Medicine, Konkuk University, Seoul, Republic of Korea; f Department of Pathobiology, College of Veterinary Medicine, Auburn University, Auburn, AL, USA

**Keywords:** Avian influenza virus, highly pathogenic avian influenza virus, clade 2.3.4.4, infectivity, pathogenicity, transmissibility, chicken

## Abstract

Clade 2.3.4.4 Eurasian lineage H5Nx highly pathogenic avian influenza virus (HPAIV) has become the globally dominant clade and caused global outbreaks since 2014. The clade 2.3.4.4 viruses have evolved into eight hemagglutinin subgroups (2.3.4.4a-h). In this study, we evaluated the infectivity, pathobiology, and transmissibility of seven clade 2.3.4.4 viruses (two 2.3.4.4a, two 2.3.4.4b, one 2.3.4.4c and two 2.3.4.4e) in chickens. The two clade 2.3.4.4e viruses caused 100% mortality and transmissibility in chickens. However, clade 2.3.4.4a and c viruses showed 80–90% mortality and 67% transmissibility. Clade 2.3.4.4b viruses showed 100% mortality, but no transmission to co-housed chickens was observed based on lack of seroconversion. All the infected chickens died showing systemic infection, irrespective of subgroup. The results highlight that all the clade 2.3.4.4 HPAIVs used in this study caused high mortality in infected chickens, but the transmissibility of the viruses in chickens was variable in contrast to that of previous Eurasian-lineage H5N1 HPAIVs. Changes in the pathogenicity and transmissibility of clade 2.3.4.4 HPAIVs warrant careful monitoring of the viruses to establish effective control strategies.

## Introduction

The Gs/GD lineage H5 highly pathogenic avian influenza virus (HPAIV) was first detected in geese from Guangdong, China, in 1996 (A/goose/Guangdong/1/1996 [H5N1], Gs/GD lineage). Since then, it has caused continuous outbreaks in enzootic countries and has evolved into multiple hemagglutinin (HA) genetic clades [1,2]. H5N1 was the dominant HA and neuraminidase (NA) subtypes of the Gs/GD lineage; however, since 2008, novel NA subtypes of Gs/GD lineage HPAIVs belonging to the third order clade 2.3.4, such as H5N2, H5N5, H5N6, and H5N8, have been identified in China and subsequently the HA evolved into additional subclades, including fourth order clade 2.3.4.4 [3–5]. In addition, clade 2.3.4.4 H5Nx has evolved into eight fifth order HA genetic subgroups (a-h) and multiple genotypes, bearing variable gene segment constellations by reassortment with low pathogenicity avian influenza viruses (LPAIVs) in domestic ducks and wild birds [3–7]. Clade 2.3.4.4 H5Nx HPAIVs have caused infections in multiple species, including poultry, wild birds, several mammalian species, and humans; thus, the virus represents a serious threat to veterinary and public health and the poultry industries [4,6–8].

Since 2014, clade 2.3.4.4 H5Nx HPAIVs have been detected in wild waterfowl and have spread worldwide, including Europe, Africa, and the Americas [4,9,10]. During 2014–2016, clade 2.3.4.4c H5N8 HPAIVs caused multiple outbreaks in various regions worldwide, including the first Gs/GD lineage HPAIV outbreak in North America [10,11]. In May–June 2016, a novel reassortant clade 2.3.4.4b H5N8 HPAIV was detected in wild birds in Qinghai Lake, China, and Uvs-Nuur Lake in Siberia. Subsequently, the virus spread to various regions in Asia, Africa, and Europe [4,12–14]. The related clade 2.3.4.4b H5Nx HPAIVs have circulated in the wild bird population, and multiple genotypes bearing gene segments of wild bird-origin LPAIVs have been detected mainly in Eurasia [12–14]. Since 2021, novel reassortment clade 2.3.4.4b H5N1 HPAIVs have caused worldwide epizootics in Europe, Africa, Asia, and the Americas [15–18]. Unlike the previous viruses, the clade 2.3.4.4b virus has been maintained in wild birds for a long time, causing changes in the epidemiology of HPAIVs [19,20].

The clades 2.3.4.4d-h of the H5N6 subtype have been predominant in China since 2014 and their geographic distribution has been limited to East Asia. Particularly, the clade 2.3.4.4e H5N6 was detected in wild birds in 2016 and subsequently caused large outbreaks in South Korea and Japan during 2016–2017 [6,21,22], with multiple reassortants being detected in both domestic poultry and wild birds [22,23]. Clade 2.3.4.4a H5N6 virus has only been detected in Asian enzootic countries since 2013, and distant geographical spread to other regions has not been reported [6,7].

Unlike previous H5N1 HPAIVs of the Gs/GD lineage that caused almost 100% mortality in chickens, clade 2.3.4.4 H5Nx HPAIVs have showed varying and relatively lower mortality rates in experimentally inoculated or contact exposed chickens [24–26]. In addition, clade 2.3.4.4 virus-infected duck species varied from asymptomatic to high mortality but shed high viral titers [25,27,28]. Most of the clade 2.3.4.4 viruses exhibited low mortality in duck species; however, an exceptional high mortality rate was observed when mallards were experimentally infected with the A/Tufted-duck/Denmark/11470/LWPL/2016 H5N8 clade 2.3.4.4b virus [27,28]. While diverse subgroups and genotypes of clade 2.3.4.4 HPAIVs have been reported, their pathogenicity in avian species have been examined for only a few strains. Furthermore, the pathogenicity and transmissibility of clade 2.3.4.4 viruses in chickens have been evaluated in different laboratories under different experimental conditions. To determine whether the biological characteristics are different for these genetically divergent clade 2.3.4.4 viruses, we evaluated the pathogenicity and transmissibility of seven H5Nx HPAIV strains representing clade 2.3.4.4 subgroups a, b, c, and e in specific pathogen free (SPF) White Leghorn chickens.

## Materials and methods

### Animals

One hundred one SPF White Leghorn chickens obtained from the U.S. National Poultry Research Center (USNPRC) in-house flocks were used. All birds were transferred at 6 weeks of age to the animal biosafety level 3 enhanced facilities and housed in negative-pressure HEPA-filtered isolators for the HPAIV challenge. The birds were provided *ad libitum* access to feed and water throughout the experiment. All procedures were performed according to protocols approved by the Institutional Laboratory Animal Care and Use Committee of the USNPRC.

### Viruses

The following seven clade 2.3.4.4 H5Nx HPAIVs were used as challenge viruses and obtained from the Southeast Poultry Research Laboratory, USNPRC HPAIV repository: A/chicken/Vietnam/NCVD14-A324/2014 (A/Ck/VNM/14; H5N6, 2.3.4.4a)(original source, Nguyen Van Long, Department of Animal Health, Vietnam), A/duck/Vietnam/NCVD-15A74/2015 (A/Dk/VNM/15; H5N6, 2.3.4.4a)(original source, Nguyen Van Long, Department of Animal Health, Vietnam), A/tufted-duck/Denmark/11740/2016 (A/TD/DNK/16; H5N8, 2.3.4.4b)(original source, Lars Erik Larsen, University of Copenhagen, Denmark), A/turkey/Hungary/53433/2016 (A/Tk/HUG/16; H5N8, 2.3.4.4b)(original source, Ian Brown, Animal and Plant Health Agency, United Kingdom), A/turkey/Minnesota/12582/2015 (A/Tk/MN/15; H5N2, 2.3.4.4c)(original source, Mia Torchetti, National Veterinary Services Laboratories, USA), A/chicken/Hokkaido/1/2016 (A/Ck/Hok/16; H5N6, 2.3.4.4e)(original source, Hiroshi Kida, Hokkaido University, Japan), and A/mandarin_duck/Korea/K16-187-3/2016 (A/MD/KOR/16; H5N6, 2.3.4.4e)(original source, Chang-Seon Song, Konkuk University, South Korea). The viruses were propagated and titrated using 9-to-11-day-old SPF embryonating chicken eggs in biosecurity level-3 enhanced facilities in the USNPRC [29].

### Experimental design and sample collection

The birds were divided into seven groups of 13 birds each for virus challenge and one group of 10 birds as a negative control. At 6 weeks of age, ten birds from each group were challenged via the intrachoanal route with approximately 10^6^ 50% egg infectious doses (EID_50_) in 0.1 mL of each isolate, and the inoculum was back titered on the day of inoculation (Table 1). To evaluate contact transmissibility, the remaining three birds per group were transferred into the isolators with the inoculated birds on 1-day post-inoculation (dpi). Negative control birds were sham-inoculated with phosphate-buffered saline (PBS).

**Table 1.** Mortality, virus shedding, and seroconversion of chickens inoculated with and contact of clade 2.3.4.4 highly pathogenic avian influenza viruses. Inoculum dose for intrachoanal inoculation included in first column.

Challenge viruses (clade) (titer^a^)	Group	Mortality (dead/total) (MDT^b^)	Viral shedding^c^ (positive/total)	Seroconversion^d^ (positive/total)
A/Ck/VNM/14 (2.3.4.4a) (10^5.7^)	Inoculated	9/10 (3.3)	9/10	0/1
	Contact	2/3 (4.5)	2/3	0/1
A/Dk/VNM/15 (2.3.4.4a) (10^5.9^)	Inoculated	9/10 (3.2)	9/10	0/1
	Contact	2/3 (4.5)	2/3	0/1
A/TD/DNK/16 (2.3.4.4b) (10^6.1^)	Inoculated	10/10 (2.1)	10/10	N/A
	Contact	0/3	0/3	0/3
A/Tk/HUG/16 (2.3.4.4b) (10^6.5^)	Inoculated	10/10 (2.1)	10/10	N/A
	Contact	0/3	0/3	0/3
A/Tk/MN/15 (2.3.4.4c) (10^5.9^)	Inoculated	8/10 (2.1)	8/10	0/2
	Contact	2/3 (3.5)	2/3	0/1
A/Ck/Hok/16 (2.3.4.4e) (10^6.3^)	Inoculated	10/10 (2)	10/10	N/A
	Contact	3/3 (3)	3/3	N/A
A/MD/KOR/16 (2.3.4.4e) (10^6.5^)	Inoculated	10/10 (2)	10/10	N/A
	Contact	3/3 (2)	3/3	N/A
Sham (PBS)	Negative control	0/10	0/10	0/10

Note: N/A: not applicable.

^a^ The viruses were inoculated via the intra-choanal route with approximately 10^6^ EID_50_ in 0.1 mL of each isolate, and the inoculum was back tittered.

^b^ MDT, Mean death time.

^c^ Number of birds shedding detectable viruses by the oral and/or cloacal route in at least one time point / total number of birds. The viral shedding was evaluated and quantified by H5 gene-specific qRT-PCR.

^d^ Determined in 14 dpi serum samples from surviving chickens by HI assay using homologous antigen.

All chickens were monitored daily for clinical signs and mortality until 14 dpi. Chickens showing severe clinical signs, which might include severe ataxia, severe lethargy, or inability to eat or drink, were euthanized and counted as dead the next day in the mean death time (MDT) calculations. Oropharyngeal (OP) swabs and cloacal (CL) swabs were collected at 1, 2, 4, 7, 10 and 14 dpi using synthetic swab on plastic stem and 1.5 mL brain heart infusion (BHI) medium (Becton, Dickinson and Company, Sparks, MD, USA) with antibiotics and antifungal (penicillin/streptomycin/amphotericin B; Hyclone, Logan, UT, USA) and stored at −80C.

Three dead or euthanized chickens from each challenge group were necropsied at 2 dpi to examine for gross lesions and to collect tissues for histological evaluation. The nasal turbinates, trachea, lungs, heart, brain, intestine, pancreas, liver, spleen, thymus, cloacal bursa, kidneys, and skeletal muscle were collected in 10% neutral buffered formalin for histopathological examination by hematoxylin–eosin (HE) staining and influenza nucleoprotein viral antigen detection by immunohistochemical (IHC) staining [30]. Other sections of the brain, heart, lungs, and spleen were collected for viral RNA detection and stored at −80C.

Serum samples were collected on the day of the challenge (0 dpi) from two birds in each group, and at 14 dpi from all surviving birds. Seroconversion against the challenge virus was evaluated by a hemagglutination inhibition (HI) assay using homologous antigens [31]. All the surviving birds were humanely euthanized by ketamine/xylazine anesthesia followed by cervical dislocation at 14 dpi.

### Virus quantification

Total viral RNA was extracted from 50 μL of the swab material using the MagMAX-96 AI/ND Viral RNA Isolation Kit (Thermo Fisher Scientific, Carlsbad, CA, USA) following the manufacturer’s instructions. Tissues were homogenized in BHI broth to a 10% solution, and total RNA was extracted from the 250 μL supernatant of tissue homogenates by RNA precipitation using the Trizol LS reagent (Invitrogen/Thermo Fisher Scientific, Grand Island, NY, USA) and the RNeasy Mini Kit (Qiagen Corp, Valencia, CA, USA). Total RNA extracted from the tissue samples was quantified using an Epoch spectrophotometer (Biotek, Winooski, VT, USA) and diluted in PBS to obtain 50 ng/µL for normalization.

Because of the low compatibility of the previous influenza virus matrix gene-targeting real-time polymerase chain reaction (PCR) system [32] for clade 2.3.4.4 viruses, the amount of influenza virus RNA was quantified by customized H5 gene-specific quantitative reverse transcription-PCR (qRT-PCR). Two primers, H5-380F (5′-AAACACCTRTTGAGCAGAATAAATCATT-3′) and H5-525R (5′-AGCCATACCACATTTCTGAA-3′), and a probe, H5-421P (5′-FAM-GATCATCCCCARGAGTTCTTGG-TAMRA-3′), were used for qRT-PCR. Quantitative real-time RT–PCR was performed using the AgPath-ID one-step RT–PCR kit (Ambion/Thermo Scientific, Grand Island, NY, USA) according to the manufacturer’s instructions.

To extrapolate the cycle threshold (Ct) values to infectious unit equivalents, the known titer of the challenge virus stocks was subjected to 10-fold serial dilutions. Viral RNA was extracted from these dilutions and run in duplicate using qRT-PCR to produce a standard curve for each challenge virus, as described above. Previous AIV challenge studies in chickens have shown a high correlation between the quantity of RNA as determined using qRT-PCR and the infectious titer [33]. The detection limit was 10^2.5^ EID_50_/mL for all viruses.

### Statistical analysis

Statistical analyses were performed using the Prism 5 software (GraphPad Software, San Diego, CA, USA). Statistical differences in mean viral titers and antibody levels among groups were analysed using one-way ANOVA with Tukey’s post hoc test. Fisher’s exact test was used to analyse the statistical significance of the number of birds shedding detectable virus titers. During virus shedding analysis, the samples not detected using qRT-PCR were treated as 10^2.4^ EID_50_/mL for statistical purposes.

## Results

### Mortality and transmissibility

The back titration results indicated that birds were inoculated with 10^5.7^–10^6.5^ EID_50_ of each clade 2.3.4.4 virus, and these viruses caused different mortality in the inoculated chickens (Table 1). All chickens inoculated with clade 2.3.4.4b viruses (A/TD/DNK/16 and A/Tk/HUG/16) and clade 2.3.4.4e viruses (A/Ck/Hok/16 and A/MD/KOR/16) died within 3 dpi. Conversely, two of the 10 chickens in the clade 2.3.4.4c virus (A/Tk/MN/15) inoculated group and one of the 10 chickens in the clade 2.3.4.4a virus (A/Ck/VNM/14 and A/Dk/VNM/15) inoculated groups survived and showed no clinical signs through the end of the experiment on 14 dpi. All chickens in the sham group survived and showed no clinical signs through 14 dpi. The shortest MDT (Table 1) was detected in the two clade 2.3.4.4e (2 days), A/Tk/HUG/16 (2.3.4.4b) (2.1 days) and A/Tk/MN/15 (2.3.4.4c) (2.1 days) virus inoculated groups, and a relatively long MDT was detected in the two clade 2.3.4.4a virus inoculated groups (3.2 and 3.3 days).

Different transmissibility rates for clade 2.3.4.4 viruses were detected in co-housed chickens (Table 1). All three chickens co-housed with clade 2.3.4.4e inoculated groups died at 3–4 days post contact. However, all three chickens co-housed with clade 2.3.4.4b-inoculated birds survived through 14 dpi. Two of the three chickens in the clade 2.3.4.4a and 2.3.4.4c virus inoculated groups died at 4–6 days post contact.

### Viral shedding and seroconversion

**Figure 1.**
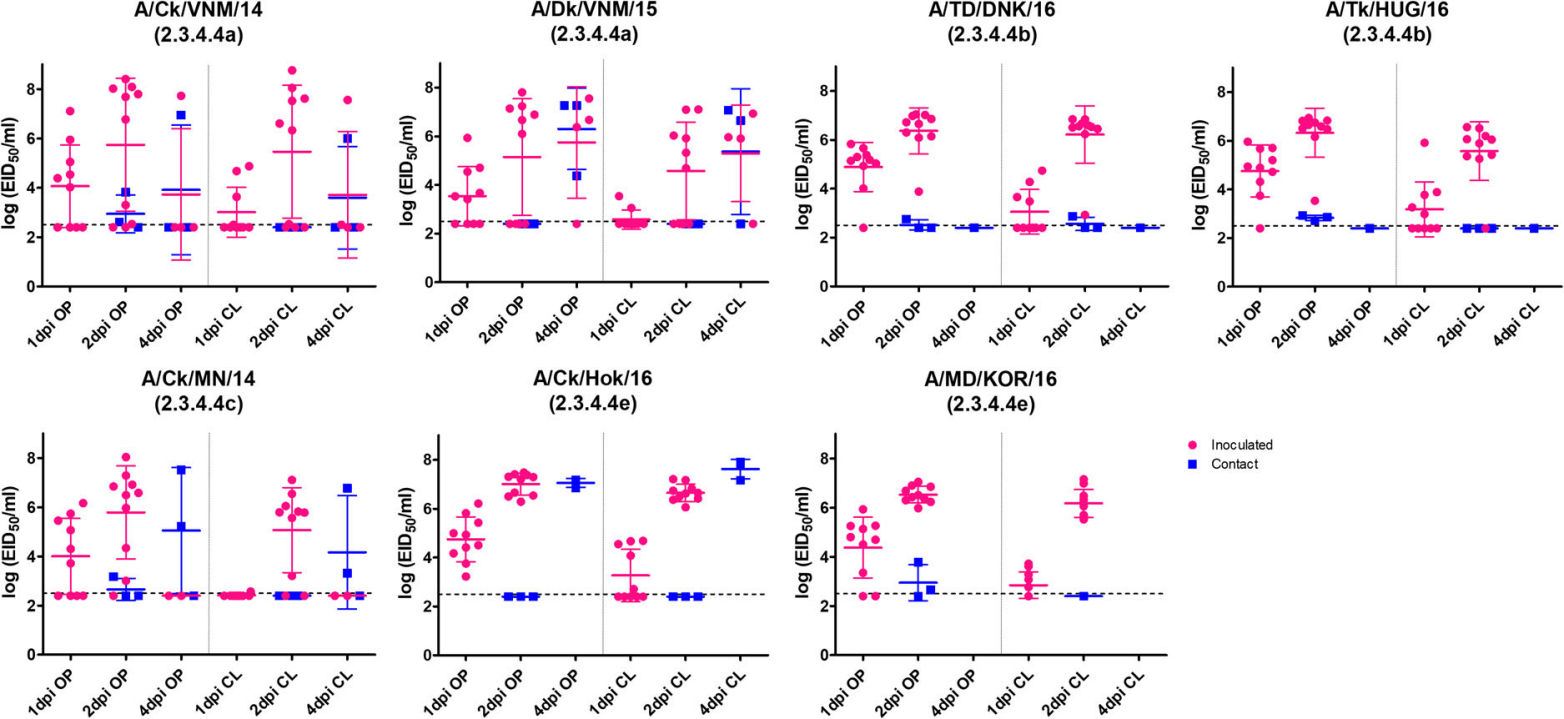
Egg infectious dose-equivalent titers from the oropharyngeal (OP) and cloacal (CL) swabs collected from chickens inoculated with or in contact with chickens shedding clade 2.3.4.4 highly pathogenic avian influenza viruses. Chickens in contact group were transferred into the isolators with the inoculated birds on 1 dpi. All samples were negative at 7, 10, and 14 dpi and are not shown. The lower detection limit was 10^2.5^ 50% egg infectious dose (EID_50_)/mL. Negative RT-PCR samples were treated as 10^2.4^ EID_50_/mL for statistical purposes.

Most dead birds had high titers of virus in the oropharynx (mean: 10^6.3^ EID_50_/mL) and cloaca (mean: 10^5.9^ EID_50_/mL) at 2 dpi (Figure 1). At 1 dpi, higher titers of virus were detected in OP swab samples (mean: 10^4.3^ EID_50_/mL) than in CL swab samples (mean: 10^2.9^ EID_50_/mL) in all groups, but statistically significant (*p* < 0.05) differences between OP and CL viral titers were only detected in clades 2.3.4.4b and 2.3.4.4e virus-inoculated groups. At 2 dpi, however, similar virus titers were detected in both OP and CL swabs.

In clade 2.3.4.4b, 2.3.4.4c, and 2.3.4.4e virus-inoculated groups, viral shedding was detected until 2 dpi, because all the infected birds were dead before 3 dpi. However, one bird in the A/Ck/VNM/14 virus-inoculated group and three birds in the A/Dk/VNM/15-inoculated group were positive for virus shedding at 4 dpi and died within 6 dpi. The peak viral shedding titers and the number of chickens shedding detectable virus did not show statistically significant differences among groups.

At 2 and 4 dpi, low viral titers (less than 10^3.0^ EID_50_/mL) were detected in the OP and CL swabs of some birds that survived until the end of experiment, probably due to mechanical virus exposure from contaminated food and water during co-housing with the infected birds, rather than due to active infection. From 7 dpi onwards, viral shedding was not detected from any surviving birds. To assess the possibility of subclinical infection of clade 2.3.4.4 HPAIVs in the surviving chickens, we evaluated the HA antibody responses to challenge viruses using the HI test. HI antibodies were not detected in any of the pre-challenge serum samples. Serum samples collected at 14 dpi were all negative, both from sham-inoculated chickens and from surviving HPAIV-inoculated chickens supporting the latter lacked active infection.

### Microscopic lesions and viral antigen distribution

**Figure 2.**
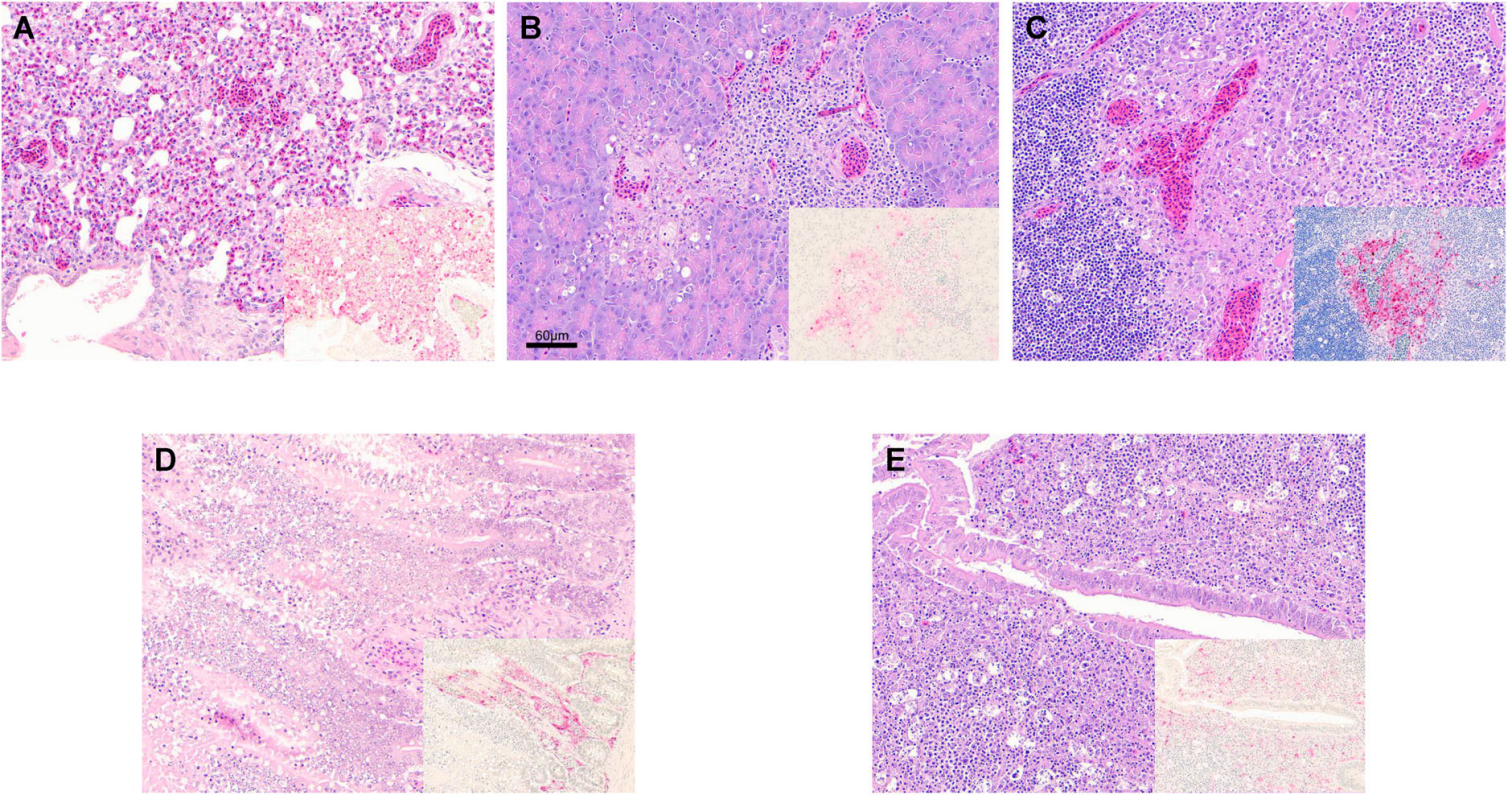
Histopathology and immunohistochemical staining for AIV antigen in tissues of chickens infected with clade 2.3.4.4 highly pathogenic avian influenza viruses, 2 days post-inoculation. Hematoxylin-eosin staining (A-E); immunohistochemistry (insets), virus staining of cells (nuclei – clearly stained circular bodies) in red. **A.** Lung, A/Ck/VNM/14 (clade 2.3.4.4a). Interstitial pneumonia with lymphoplasmacytic infiltrates. Inset: Lung, same area. Viral antigen in endothelial cells, pneumocytes, mononuclear cells. **B.** Pancreas, A/TD/DNK/16 (clade 2.3.4.4b). Necrotic pancreatic acinar cells with lymphoplasmacytic infiltrates. Inset: Pancreas, same area. Viral antigen in acinar cells, duct cells, endothelial cells. **C.** Thymus, A/TD/DNK/16 (clade 2.3.4.4b). Necrosis in medulla and lymphocyte depletion in cortex. Inset: Thymus, same area. Viral antigen in mononuclear cells, thymic epithelium in medullary area, endothelial cells. **D.** Duodenum, A/Ck/Hok/16 (clade 2.3.4.4e). Focal necrosis with lymphoplasmacytic infiltrates in submucosa. Inset: Duodenum, same area. Viral antigen in enterocytes, endothelial cells, mononuclear cells in lymphoid associated tissue, smooth muscle cells. **E.** Cecal tonsil, A/MD/KOR/16 (clade 2.3.4.4e). Focal necrosis with lymphoplasmacytic infiltrates in submucosa. Inset: Cecal tonsil, same area. Viral antigen in enterocytes, endothelial cells, mononuclear cells in lymphoid associated tissue.

Collectively, similar types of histological lesions and types of cells expressing virus antigen were observed in infected chickens with all the viruses tested (Tables 2 and 3, Figure 2). The most prominent lesion across viruses and tissues was necrosis of parenchymal cells, often accompanied by lymphoplasmacytic or heterophilic inflammatory infiltrates (Table 3). Viral antigen was consistently detected in parenchymal cells and vascular endothelial cells of most tissues, regardless of the challenge virus, in nasal tissues, lung, heart, brain, spleen, and thymus, which were the most affected tissues. Marked lesion heterogeneity in immune tissues (spleen, cloacal bursa, and thymus) was observed among birds. In these tissues, lymphoid depletion due to necrosis was common, although in some cases, viral antigen was widespread in distribution, indicating active replication at the time of necropsy. In other cases, no viral staining could be observed, suggesting active replication prior to necropsy.

**Table 2.** Microscopic lesions and viral antigen distribution in chickens inoculated with clade 2.3.4.4 viruses, 2 days post-inoculation.

	A/Ck/VNM/14 (2.3.4.4a)		A/Dk/VNM/15 (2.3.4.4a)^a^		A/TD/DNK/16 (2.3.4.4b)		A/Tk/HUG/16 (2.3.4.4b)		A/Tk/MN/15 (2.3.4.4c)		A/Ck/Hok/16 (2.3.4.4e)		A/MD/KOR/16 (2.3.4.4e)	
Tissue	HE^b^	IHC^c^	HE	IHC	HE	IHC	HE	IHC	HE	IHC	HE	IHC	HE	IHC
Nasal turbinates	++ − ++	+++ + +++	++ ++	+++ +++	++ ++ +++	+++ ++ +++	− +++ +++	+ +++ +++	++ ++ ++	+++ ++ +	++ ++ ++	+++ +++ +++	++ ++ ++	+++ ++ +++
Trachea	− + −	+ ++ −	− −	− −	nt ++ ++	nt ++ ++	− − −	+ − −	− − −	++ − +	− ++ −	+ +++ −	− − ++	+ + ++
Lung	+ +++ +++	++ +++ +++	+++ +++	+++ +++	++ ++ +++	++ +++ +++	+++ +++ +++	+++ +++ +++	+++ ++ +++	++ ++ +++	− − −	− − ++	+ + ++	++ ++ +++
Heart	+++ +++ +++	+++ +++ +++	+++ −	+++ −	+++ − +++	+++ − +++	+++ +++ +++	+++ +++ ++	+++ +++ +++	+++ +++ +++	+++ +++ +++	+++ +++ +++	+++ +++ +++	+++ +++ +++
Brain	++ +++ ++	++ +++ +++	+++ ++	+++ +++	++ − ++	+++ + +++	− + −	++ ++ ++	+ + +	++ + +	++ ++ ++	++ +++ ++	++ ++ +	++ ++ +
Duodenum	− − −	+ +++ +++	− −	+ ++	− − +	+ ++ +++	− − −	++ ++ −	nt − −	nt + −	− + +	++ +++ +++	+ − ++	+++ ++ +++
Ileum	nt − −	nt ++ ++	− −	+ −	++ ++ +	+++ ++ +	− − −	++ − −	nt − nt	nt + nt	− − −	− ++ ++	− − nt	− +++ nt
Cecal tonsil	nt − −	nt ++ ++	− −	+ −	nt nt nt	nt nt nt	− − −	+ ++ ++	nt − −	nt ++ −	− − −	++ +++ −	− +++ −	+++ +++ ++
Pancreas	− − +	+ ++ ++	− −	++ −	++ − ++	++ − +	+ − −	++ ++ −	− − −	− + −	− − +	++ ++ ++	− − −	+ − +
Liver	− − +	− +++ +++	− −	+ −	− − −	+ + ++	− − −	+ − −	− − −	+ − −	+ + +	+++ ++ +++	− − −	++ ++ ++
Spleen	++ ++ ++	++ ++ +++	++ ++	+++ +++	+++ ++ +++	− − +++	+++ +++ +++	+++ +++ ++	++ ++ ++	+++ ++ ++	nt +++ −	nt +++ ++	− +++ +++	++ +++ +++
Thymus	+ +++ +++	++ +++ +++	+++ +++	+++ +++	− +++ +++	+ +++ +++	+++ +++ +++	+++ +++ +++	+++ − +	+++ + ++	− ++ +	− +++ −	− − −	− + +
Cloacal bursa	+ +++ +++	− +++ ++	++ ++	+++ ++	+++ +++ +++	− − +	+++ +++ −	+ − −	+ + −	+ + −	++ − −	++ + +	− +++ −	− ++ +
Kidney	− − ++	− ++ +++	− −	++ +	+ − ++	+ + ++	++ − −	+ + +	− − −	− − −	+ − −	++ − −	− − −	− + −
Skeletal muscle	− − −	− − −	− − −	− − −	− − −	− − −	− − −	− − −	− − −	− − −	− − −	− − −	− − −	− − −

Note: nt: no tissue.

^a^ One bird from the A/Dk/VNM/15-inoculated group showed no microscopic lesions or IHC staining in any tissue. Because this bird had no virus shedding or PCR-positive tissues, it was considered non-infected and was excluded from the histopathological evaluation.

^b^ Histopathology score of lesions in hematoxylin-eosin (HE) staining: −: no lesions; +: mild; ++: moderate; +++: severe.

^c^ Immunohistochemical (IHC) staining: −: no antigen staining; +: infrequent; ++: common; +++: widespread.

**Table 3.** Microscopic lesions and viral antigen distribution in chickens inoculated with clade 2.3.4.4a-e highly pathogenic avian influenza viruses, 2 days post-inoculation.

Tissue	Lesions	Cell types expressing virus antigen
Nasal cavity	Epithelial cell necrosis and desquamation, rhinitis, sinusitis, mononuclear cell infiltrates, heterophilic rhinitis	Vascular endothelial cells, vascular endothelial cells of mucosal glands, nasal epithelial cells, respiratory epithelial cells, nasal gland epithelium, mononuclear cells
Trachea	Focal necrosis, lymphoplasmacytic infiltrates	Pseudostratified epithelial cells, vascular endothelial cells, sternotrachealis muscle
Lung	Interstitial pneumonia, edema, congestion, necrosis, lymphoplasmacytic infiltrates	Vascular endothelial cells, pneumocytes, mononuclear cells
Heart	Focal necrosis of myocardiocytes	Myocardiocytes, vascular endothelial cells
Brain	Neuronal necrosis, gliosis, chromatolysis of Purkinje cell layer	Neurons, Purkinje cells, ependymal cells, glial cells, vascular endothelial cells
Intestine	Focal necrosis, lymphoplasmacytic infiltrates in submucosa	Villi enterocytes, vascular endothelial cells, mononuclear cells in lymphoid associated tissue, smooth muscle cells
Pancreas	Degeneration of individual pancreatic acinar cells, focal lymphoplasmacytic infiltrates	Acinar cells, duct cells, vascular endothelial cells
Liver	Focal necrosis, lymphoplasmacytic infiltrates	Kupffer cells, hepatocytes, vascular endothelial cells, macrophages
Spleen	Multifocal necrosis, hemorrhages, lymphoid depletion	Mononuclear cells, vascular endothelial cells
Thymus	Focal to multifocal necrosis in medullary area (evident in Hassall's corpuscles), lymphocyte depletion, apoptotic lymphocytes in cortex area	Mononuclear cells, thymic epithelium in medullary area, vascular endothelial cells
Cloacal bursa	Lymphocyte necrosis and apoptosis, lymphocyte depletion, phagocytic hyperplasia	Mononuclear cells, medullary support cells, vascular endothelial cells
Kidney	Focal necrosis of tubular epithelium with lymphoplasmacytic infiltrates	Tubular epithelial cells, glomerular cells, vascular endothelial cells
Skeletal muscle	No lesion	No lesion

Some differences in the severity of lesions and intensity of viral staining were observed among the viruses (Table 2). The intestine (duodenum, ileum, caecal tonsil) of clade 2.3.4.4a (A/Dk/VNM/15) and clade 2.3.4.4c infected chickens was unaffected or only showed mild viral antigen staining, whereas clade 2.3.4.4a (A/Ck/VNM/14), 2.3.4.4b, and 2.3.4.4e viruses replicated better in villi enterocytes and capillaries, often causing focal areas of necrosis with lymphoplasmacytic infiltrates. Viral staining in the liver was mild and infrequent with clade 2.3.4.4a (A/Dk/VNM/15), 2.3.4.4b, and 2.3.4.4c viruses but was more widespread in clade 2.3.4.4e and 2.3.4.4a (A/Ck/VNM/14) viruses. Although virus staining in vascular endothelial cells was frequent for all viruses, clade 2.3.4.4a and e viruses showed especially extensive vascular endothelial cell tropism.

**Figure 3.**
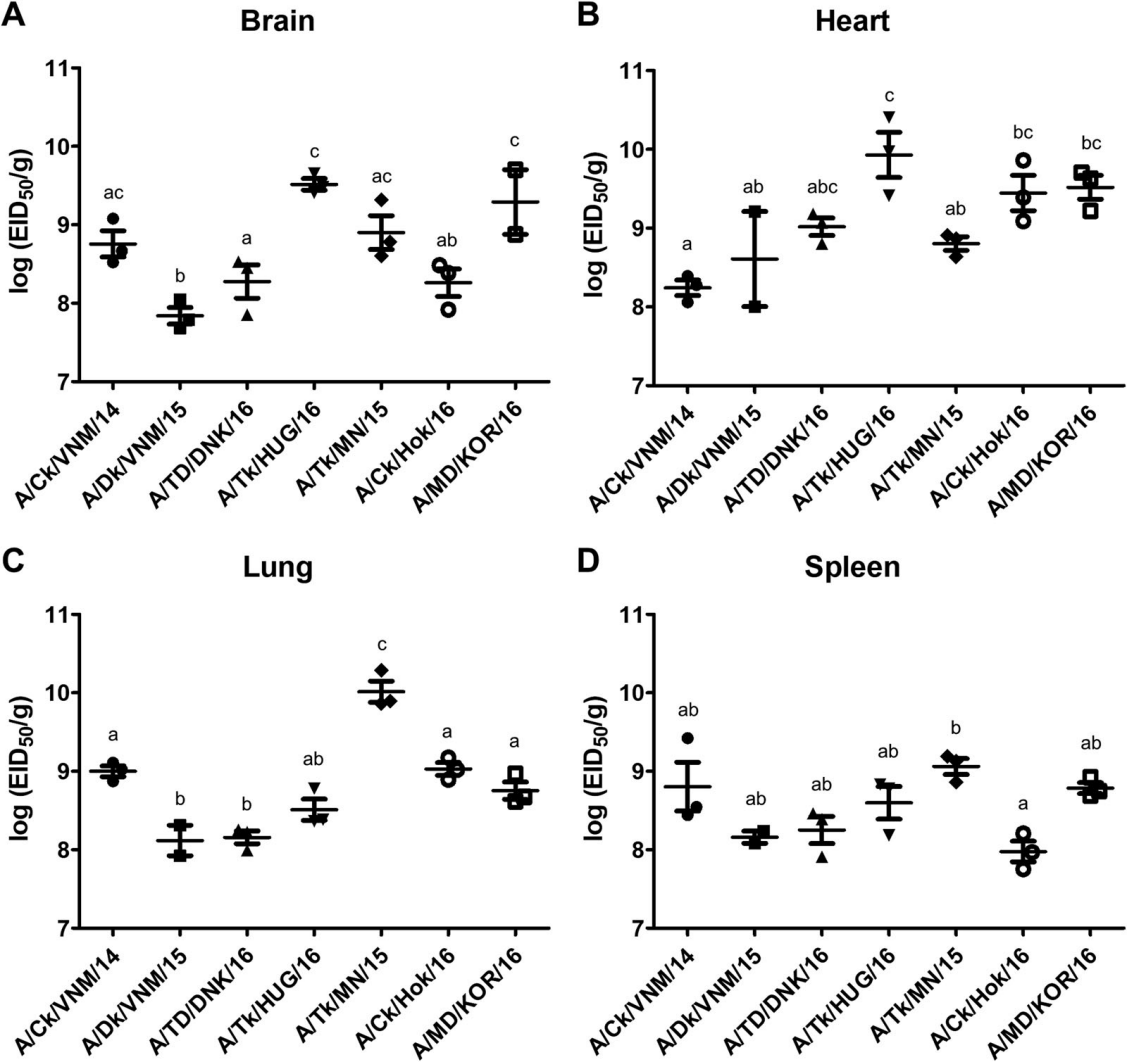
Egg infectious dose-equivalent titers in brain (A), heart (B), lung (C) and spleen (D) collected from chickens inoculated with clade 2.3.4.4 highly pathogenic avian influenza viruses at 2 days post-inoculation (*n* = 3 chickens/virus). Viral titers sharing at least one superscript letter indicate no statistically significant differences (*p* ≥ 0.05).

To evaluate the systemic replication of clade 2.3.4.4 viruses, viral titers in the brain, heart, lungs, and spleen were determined (Figure 3). Clade 2.3.4.4 virus-infected chickens showed high viral titers (>10^7.6^ EID_50_/g) in all four tissues except one chicken in the A/Dk/VNM/15-challenged group. The highest viral titer was detected in the lungs of the clade 2.3.4.4c virus challenge group and the heart of the clade 2.3.4.4b challenge group. The clade 2.3.4.4e challenge groups showed high viral titers in the brain and heart. For clade 2.3.4.4a viruses, A/Ck/VNM/14-challenged chickens showed high titers in the lungs and spleen, but A/Dk/VNM/15-challenged chickens showed high titers in the brain and heart.

Viral antigen and RNA were not detected in any tissue of one chicken of A/Dk/VNM/15-challenged group. This chicken was euthanized for tissue sample collection at 2 dpi, and viral shedding was not detected, indicating lack of successful HPAIV infection.

## Discussion

Clade 2.3.4.4 H5Nx viruses emerged by gradual mutations and multiple reassortment between H5N1 HPAIVs and LPAIVs in ducks [3–6]. Previous studies have shown that these viruses can efficiently replicate in duck species without causing high mortality, indicating that clade 2.3.4.4 HPAIVs are highly adapted to duck species [25,34,35]. Other studies have suggested that some clade 2.3.4.4 HPAIVs show relatively low infectivity and transmissibility in chickens compared to previous H5N1 viruses of the Gs/GD lineage, which typically show 100% mortality in chickens [24,26,36]. In this study, clades 2.3.4.4a and 2.3.4.4c HPAIVs showed 80–90% mortality in chickens. All surviving chickens lacked seroconversion, indicating no active infection despite co-housing with infected birds that shed high viral titers. A previous infectivity study identified high 50% chicken infectious doses (10^5.7^ and 10^4.4^ EID_50_/mL) and lack of seroconversion in surviving chickens inoculated with clade 2.3.4.4c H5N8 and H5N2 viruses, indicating lower infectivity of chickens to these clade 2.3.4.4 viruses compared to previous Gs/GD HPAIVs [24]. In addition, less than 100% transmissibility was identified in clade 2.3.4.4a-, b-, and c-exposed chickens in the present study. The difference in the mortality and transmissibility of viruses was not statistically supported because of the low number of birds, but less than 100% mortality and transmissibility are uncommon in other clades of Gs/GD lineage HPAIVs, including clade 2.3.2.1 and clade 2.2 [37,38]. All viruses used in this study caused systemic infections and lesions in the infected chickens. These results highlight that the mortality rate of clade 2.3.4.4 viruses in chickens is still high, but the susceptibility of chickens to become infected depends on the viral strain, the exposure dose and the virus’s degree of adaptation to chickens. In 2014–2025, the 2.3.4.4c clade viruses increased adaptation to chickens and turkeys after they circulated on farms, decreasing the dose of virus needed to produce infection and increased the ability to spread between farms by lateral transmission [24,25].

The viruses belonging to the same subgroup showed similar infectivity and transmissibility in this study. However, these biological characteristics can be altered during viral evolution and adaptation. For example, during the 2014–2015 clade 2.3.4.4c H5N2 epizootic in the USA, the viruses isolated from poultry during spring 2015 showed better adaption and higher infectivity in chickens, as evident by lower 50% chicken infectious dose, than the initial H5N2 virus isolated from a wild duck [25,39]. The clade 2.3.4.4b viruses used in this study were isolated from wild birds during the initial phase of the outbreak. Because the clade 2.3.4.4b viruses have caused ongoing worldwide outbreaks since 2016 until now, as of January 2023, the changes in molecular and biological characteristics of this clade should be continuously monitored. Notably, in this study, the clade 2.3.4.4e virus isolated from a wild mandarin duck during the initial outbreak showed high mortality and transmissibility in chickens without preadaptation. The poultry isolates of clade 2.3.4.4a and 2.3.4.4c viruses showed less than 100% mortality and transmissibility in this study.

The viruses used in this study had H5 genes belonging to different clade 2.3.4.4 subgroups of the Gs/GD Eurasian lineage, and multiple gene constellations with the other seven gene segments because of reassortment with LPAIVs [12,21,22,40]. For example, clade 2.3.4.4b H5N8 viruses have wild bird LPAIV origin gene segments, except for the HA, NA, and non-structural (NS) genes [12]. Additionally, the clade 2.3.4.4c H5N2 virus has North American wild bird LPAIV origin polymerase basic 1 (PB1), nucleoprotein (NP), and NA gene segments [9]. Clade 2.3.4.4a and 2.3.4.4e H5N6 viruses also emerged by multiple reassortments with previous H5N1 HPAIVs and duck-origin LPAIVs [6]. Due to these multiple reassortments, the genetic mutations that contribute to the pathogenicity changes cannot be defined in this study. Although the role of each gene segment in pathogenicity in chickens or ducks has not yet been fully determined, previous studies have shown that internal gene segments, including polymerase complex genes, can contribute to changes in virus replication and pathogenicity in chickens and ducks [39,41,42]. The clade 2.3.4.4 viruses are continuously evolving into multiple genotypes by reassortment with prevailing LPAIVs in wild birds [6,12,13,15,16,43]. Although the clade 2.3.4.4e viruses showed higher mortality and transmissibility than other viruses in this study, the pathogenicity of a specific virus can be readily changed by further reassortment with other viruses. Therefore, the biological characteristics of newly emerging genotypes should be continuously monitored to inform veterinary authorities of any increases in adaptation to poultry and thus an increased risk of transmission.

Clade 2.3.4.4 H5Nx HPAIVs have become the dominant clade in wild birds since 2014, especially the clade 2.3.4.4b HPAIVs which have caused global outbreaks in Asia, Europe, Africa, and America [4,8,10,14–18,43–45]. The relatively low infectivity and transmissibility of some clade 2.3.4.4 H5Nx viruses for chickens, especially broiler chickens, compared to previous Gs/GD-lineage H5N1 HPAIVs may delay and hinder rapid, accurate diagnoses on farms and may limit early disease control. Sharing updated pathogenicity information with field veterinarians and poultry farmers will be crucial for the early detection and control of HPAIVs.

During the 2016–2017 clade 2.3.4.4b HPAIV epizootic in Europe, most poultry cases were detected in domestic waterfowl, including ducks and geese, and outbreaks were focused in high duck density areas such as France and Hungary [46]. In South Korea, domestic ducks played a central role in the clade 2.3.4.4c H5N8 outbreak of 2014–2016; however, clade 2.3.4.4e H5N6 outbreaks occurred mostly in chickens [47,48]. Our results showed that clade 2.3.4.4e H5N6 viruses caused higher mortality and transmissibility than other clade 2.3.4.4 viruses in chickens, in line with field observations where clade 2.3.4.4e viruses predominated in chicken premises. Understanding the pathogenicity and susceptibility of poultry species to novel HPAIVs is a key to predicting disease outbreak patterns and establishing targeted control strategies.

We identified different levels of infectivity and transmissibility rates, and pathobiology of clade 2.3.4.4 H5Nx subgroups. In contrast to previous H5N1 HPAIVs of the Gs/GD lineage, clade 2.3.4.4 H5Nx HPAIVs continuously cause infection in wild birds and poultry, and evolve by reassortment with diverse LPAIVs in wild birds which can result in changes in pathobiology of viruses [12,15,41]. The evolution of HPAIVs and changes in their biological characteristics need to be continuously monitored for the effective control of HPAIV outbreaks.

## Supplementary Material

Supplemental Material
