# Supplementary figures and images for: Diverse infectivity, transmissibility, and pathobiology of clade 2.3.4.4 H5Nx highly pathogenic avian influenza viruses in chickens
Source: Emerg Microbes Infect. 2023 Jun 12;12(1):2218945. doi: 10.1080/22221751.2023.2218945 (PMC10262800; doi:10.1080/22221751.2023.2218945)

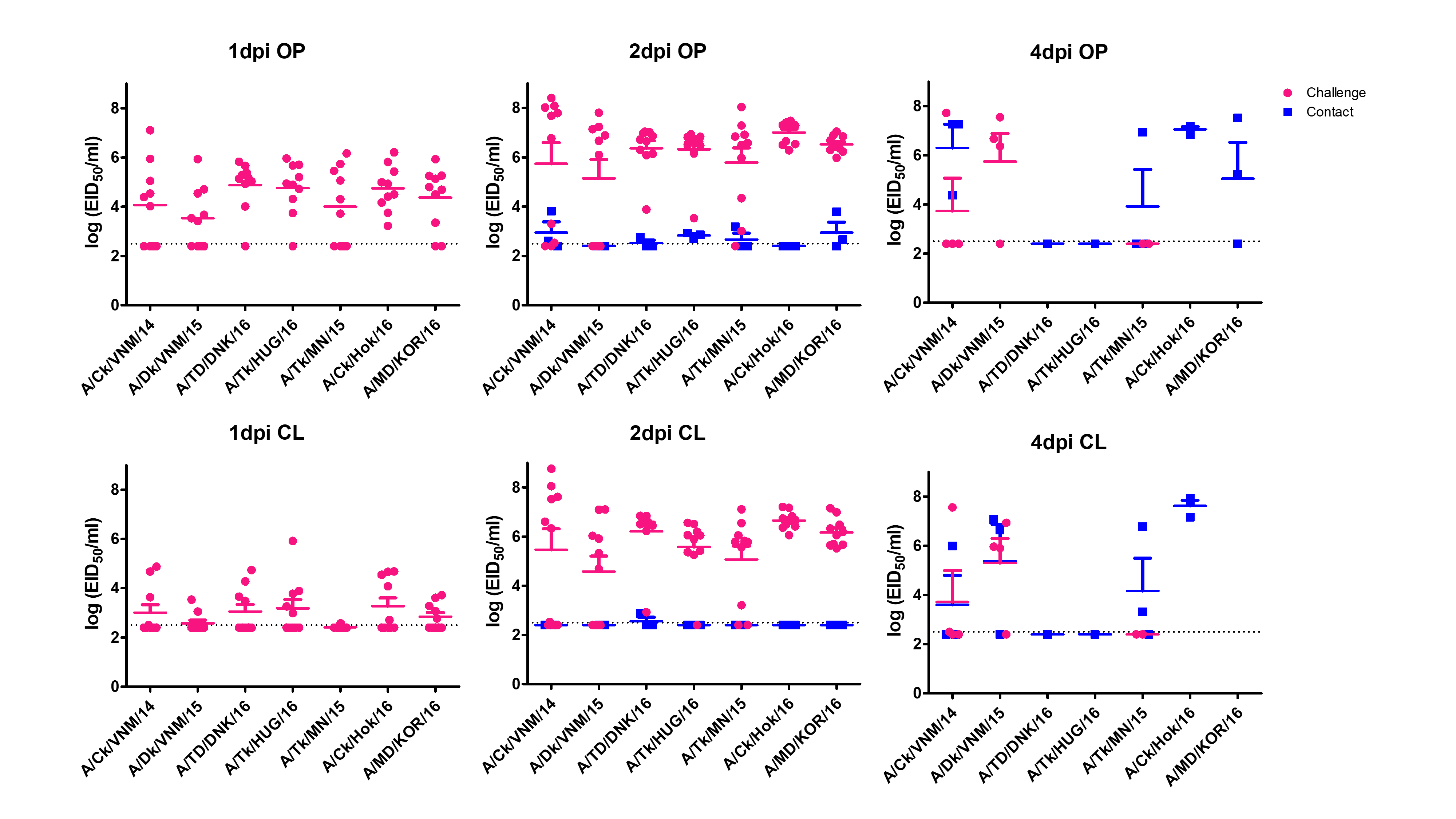

Supplement: Supplemental Material [file TEMI_A_2218945_SM5694.tif]
